# The Effects of 6 Months of Progressive High Effort Resistance Training Methods upon Strength, Body Composition, Function, and Wellbeing of Elderly Adults

**DOI:** 10.1155/2017/2541090

**Published:** 2017-06-06

**Authors:** James Steele, Kristin Raubold, Wolfgang Kemmler, James Fisher, Paulo Gentil, Jürgen Giessing

**Affiliations:** ^1^School of Sport, Health, and Social Sciences, Southampton Solent University, Southampton, UK; ^2^Institute of Sport Science, University of Koblenz-Landau, Landau, Germany; ^3^Faculty of Physical Education and Dance, Federal University of Goias, Goiania, GO, Brazil; ^4^Institute of Medical Physics, Friedrich-Alexander University, Erlangen-Nürnberg, Erlangen, Bavaria, Germany

## Abstract

**Purpose:**

The present study examined the progressive implementation of a high effort resistance training (RT) approach in older adults over 6 months and through a 6-month follow-up on strength, body composition, function, and wellbeing of older adults.

**Methods:**

Twenty-three older adults (aged 61 to 80 years) completed a 6-month supervised RT intervention applying progressive introduction of higher effort set end points. After completion of the intervention participants could choose to continue performing RT unsupervised until 6-month follow-up.

**Results:**

Strength, body composition, function, and wellbeing all significantly improved over the intervention. Over the follow-up, body composition changes reverted to baseline values, strength was reduced though it remained significantly higher than baseline, and wellbeing outcomes were mostly maintained. Comparisons over the follow-up between those who did and those who did not continue with RT revealed no significant differences for changes in any outcome measure.

**Conclusions:**

Supervised RT employing progressive application of high effort set end points is well tolerated and effective in improving strength, body composition, function, and wellbeing in older adults. However, whether participants continued, or did not, with RT unsupervised at follow-up had no effect on outcomes perhaps due to reduced effort employed during unsupervised RT.

## 1. Introduction

The age associated decline in physical function and condition is widely evidenced. For example, bone mineral density, muscle mass, strength, and cardiorespiratory fitness all decline with increasing age and affect health and wellbeing [1–6]. The World Health Organisation physical activity guidelines for older adults including a range of approaches are designed to attenuate this age-related decline [7]. In particular, due to the loss of muscle mass and strength, inclusion of whole body “muscle strengthening activities” (e.g., resistance training: RT) is encouraged 2x/week.

Participation in RT is associated with reduced morbidity and mortality risk in the elderly [8, 9]. Hurley and Roth [10] noted that* “~2 decades of age-associated strength loss can be regained in ~2 months of resistance exercise”* and RT can even enhance cardiorespiratory fitness in older adults [11, 12]. Higher levels of strength and cardiorespiratory fitness are also associated with greater cognitive function [13, 14] as well as functional ability, improved walking speed, and a reduced risk of falling [15–19]. The outcomes of RT, such as increased muscle mass [20, 21], strength [22–26], and cardiorespiratory fitness [27, 28], may even contribute to reduced mortality risk in the elderly. Further, though there is interindividual variability in responsiveness in older adults [29], all seem to benefit in some way from RT [30].

In consideration of the benefits of RT in older adults, studies have examined the manipulation of RT variables (length of training intervention, load, repetition range, repetition duration, rest periods, training frequency, and set volume) for optimal benefits [31]. Indeed, recent meta-analyses have attempted to characterise the literature in this regard [32, 33]. These generally highlight the fact that a range of RT approaches seem similarly effective for older adults reporting large effect sizes (ES). However, one variable often not considered is the role of effort and as such the set end points used during RT, a variable that in younger adults has been suggested to potentially impact upon adaptation [34, 35].

Clear definitions of set end points in RT, representing a progression of intensity of effort, have recently been suggested including: nonrepetition maximum (nRM), self-determined repetition maximum (sdRM), momentary failure (MF), and momentary failure plus advanced techniques (MF+) [36]. In these the nRM represents completion of an arbitrary predetermined number of repetitions despite a person being able to perform more, the sdRM represents the point where a person determines they could not complete the next repetition if it were attempted (i.e., they predict MF on the next repetition), MF represents the point where a person cannot complete the current repetition in the prescribed form despite attempting to do so, and MF+ is where after reaching MF a person continues using an advanced RT technique such as forced repetitions or drop sets. Within these definitions MF represents the point of maximal effort as it is the point where, despite the greatest effort, a person is unable to meet and overcome the demands of the exercise.

Intensity of effort may be important in determining the efficacy of RT in older adults. Where nRM has previously been used as set end point (a target repetition number in combination with a submaximal rating of perceived exertion [RPE] using the OMNI-RES Scale) there were no significant improvements in any outcome measures compared to a nontraining control group over 8 weeks of supervised elastic band based RT [37]. One recent study has employed the use of sdRM as a set end point in a supervised low volume (single set) and low frequency (twice a week) RT intervention [38] reporting significant increases in strength outcomes with large within-participant ESs (1.59 to 3.31). Thought not representing a maximal effort, the application of sdRM in older adults does induce a relatively high perceived effort [39]. Considering that in younger adults there may be additional benefit of training to maximal effort (i.e., MF) it is of interest to examine higher effort approaches in older adults.

High effort RT interventions performed to MF in older adults are uncommon but have been employed previously examining the role of load [40]. Adaptations to heavier- or lighter-loads seem similar when repetitions are performed to MF in older adults [40] similarly to findings in both adolescent [41] and young adult populations [42]. However, though supervised high effort RT is effective, Van Roie et al. [43] have reported that long term adherence after the initial supervised intervention, whether using heavier- or lighter-loads, is poor. Further, the training effort of participants may drop considerably over this period. Indeed participants were performing a lower number of repetitions with training loads lighter than during the initial 12-week intervention [43]. However, measures of fitness or function were not made at follow-up. Though a relatively low dose of RT is needed to maintain strength and muscle mass after an initial 12-week RT intervention for older adults [44], it is possible that the reduced effort employed might mean that initial efforts are potentially wasted as adaptations may not be maintained.

It may be that a longer initial supervised RT intervention combined with the use of progressive introduction to high effort set end points could result in greater long term adherence and maintenance of initial adaptations. High effort RT can cause discomfort [39, 45] which could generate negative affect. Introduction to RT initially at lower efforts might permit expectations of positive outcome affect reinforcing behaviour and allowing gradual introduction to higher effort RT [46]. However, to the authors' knowledge no study has examined the application of RT in older adults using clearly defined set end points inducing progressively higher efforts. As such, the aim of the present study was to examine the progressive implementation of a high effort RT approach [47, 48] in older adults over 6 months and through a 6-month follow-up on strength, body composition, function, and wellbeing of older adults.

## 2. Materials and Methods

### 2.1. Study Design

A single arm prospective trial was conducted examining the effects of a 6 month supervised RT intervention with progressive implementation of high effort set end points in older adults. Upon completion of the 6-month intervention participants self-selected whether they continued participating in RT unsupervised or not. Participants were followed up 6 months after intervention. The study design was ethically approved by the author's institution. All procedures were performed in accordance with the ethical standards of the Helsinki Declaration. Written informed consent was obtained from all participants. The trial was registered in the ISRCTN registry (ISRCTN15170767).

### 2.2. Participants

Power analysis of effect sizes from recent meta-analysis of RT research with untrained older participants [33] was conducted to determine participant numbers (*n*) using ESs of  1.57 for improvements in strength. Participant numbers were calculated using G^*∗*^Power [49, 50]. These calculations suggested only ~3 participants were required to meet the required power of 0.8 at an alpha value of *p* < 0.05 for the statistical analyses proposed (see below). However, though this might be the minimum participant requirement for the studies primary outcome (strength) attempts were made to recruit a greater number of participants considering estimated attrition rates of potentially ~50%. A total of 28 participants were initially recruited (females *n* = 14, males *n* = 14; age 69.1 ± 4.9 years, range 61 to 80 years). Participants were required to be at least 60 years of age, to present with a medical certificate verifying their otherwise good health, to have not previously engaged in RT, and to have not any contraindication to participation in RT. Participants were excluded if they had a pacemaker (due to the use of bioelectrical impedance analysis), failed to attend ≥4 training sessions, or did not meet the above criteria. Twenty-three participants completed the study with 5 drop-outs (females *n* = 4, males *n* = 1) for unrelated health reasons. At follow-up 13 participants had continued engaging in the RT intervention unsupervised (females *n* = 5, males *n* = 8).

### 2.3. Materials and Equipment

**Figure 1:**
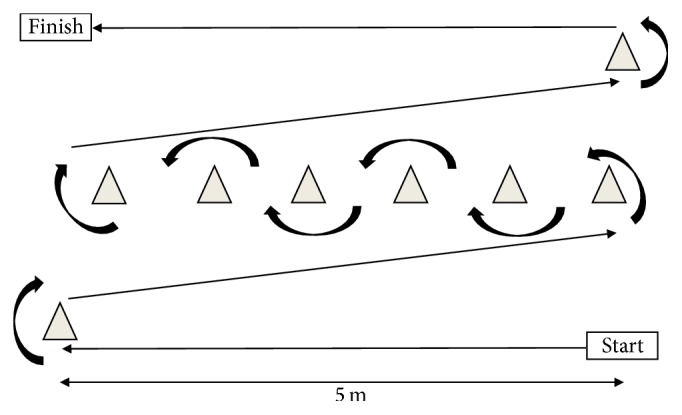
Twenty-five-metre course for carrying task.

Strength measurements and training were performed using leg press, chest press, seated row, knee extension, knee flexion, trunk extension, and trunk flexion resistance machines (Ergo-Fit, Germany). Body composition including body mass, whole body muscle, and fat mass and percentage was estimated using bioelectrical impedance (Tanita MC 180, Tanita Europe B. V., Amsterdam). This device is reported as valid compared with dual energy X-ray absorptiometry for estimating body composition in healthy adults [51]. Physical function in tasks of daily living was measured as isometric grip strength performed using a digital handgrip dynamometer (Trailite, Germany), a stair climb task involving 6 flights of 17 steps (102 steps in total) each at 17 cm height, a carrying task using a shopping basket weighing either 5 kg for females or 10 kg for males which was lifted from the ground, carried around a 25 m course involving various turns of both 90° and 180° (Figure 1), and then placed on the ground again at the end, and a chair rise task using a chair at 45 cm height. Resting heart rate was measured using an A300 Polar Monitor (Polar, Finland). Rating of perceived exertion (RPE) using Borg's CR-10 scale was taken during both the stair climb task and carrying task. Questionnaires were also used to examine perceived function and wellbeing. The WHO-5 Wellbeing Index was completed in addition to a questionnaire asking participants to rate their overall present state of health, comparison with other older adults of their age, present sporting condition, and ease with which they can perform household chores, stair climbing, shopping, gardening, and transport. For these participants were asked to provide ratings using a 5-point Likert scale ranging from 1 (“very bad”) to 5 (“very well”) for state of health, comparison with other older adults, and sporting condition and 0 (“this is very difficult for me”) to 5 (“I can manage this easily”) for ease with which they can perform household chores, stair climbing, shopping, gardening, and transport.

### 2.4. Testing

Testing was conducted before and after the intervention and at 6-month follow-up for all outcomes with the exception of physical function tests which were conducted before and after intervention only. Muscular strength testing was performed in the following order with 2-3 minutes of rest between exercises: leg press, chest press, and seated row. Participants performed a ~5RM test following National Strength and Conditioning Association guidelines for RM testing [52]. From this, predicted 1RM was calculated using the Brzycki [53] formula. Body composition was measured on a separate day from muscular performance testing both before and after the intervention following the manufacturer's guidelines. Testing for the stair climb task, carrying task, and chair rise task was performed using a stopwatch. For each the participants were instructed to begin on the command “Go” and to complete the task as quickly as possible. For the stair climb task this involved climbing the 102 steps, for the carrying task this involved picking up the shopping basket, completing the 25 m course, and then placing the shopping basket on the ground at the end of the course, and for the chair rise task this involved the participants beginning seated on the chair with their arms folded across their chest and then standing from the chair until their legs were straight five times. Isometric grip strength was taken as the average of two maximal voluntary isometric efforts. Participants positioned their arms adducted at their sides with elbows at 90° as recommended by the American Society of Hand Therapists [54]. Participants were instructed to squeeze the handle of the dynamometer progressively harder culminating in a maximal voluntary effort after 3 seconds and lasting for a further 2 seconds.

### 2.5. Training

During the intervention period training was supervised and conducted 2x/week (at least 48 hours between sessions) for 6 months (25 weeks). Participants all performed a general warm-up using either treadmill, cross-trainer, upright cycle ergometer, or recumbent cycle ergometer depending on preference for 10 minutes followed by a single set of moderate load leg press, chest press, and seated row exercises for 15 repetitions prior to each training session. In each training session participants performed leg press, chest press, seated row, knee extension, knee flexion, trunk extension, and trunk flexion. Order of exercises was not fixed and dependent upon preference and availability of equipment in the gym where training was conducted. Rest between exercises lasted for 2–4 minutes. Participants were instructed to perform the exercises using relatively long repetition duration of at least 2 seconds concentric, 1 second pause at the top of the range of motion, and 2 seconds eccentric and to not exceed 4 seconds concentric, 1 second pause at the top of the range of motion, and 4 seconds eccentric. The first 2 weeks of the intervention was a familiarisation phase whereby participants trained using a single set of each exercise using a moderate load and performing 15–18 repetitions, that is, nRM. After this period participants progressed for a further 2 weeks to perform each exercise to a set end of point of sdRM defined as cessation at the point where participants predicted they would reach momentary failure if the next repetition was attempted [36]. After this period participants progressed to perform each exercise to a set end point of MF and continued training in this manner until week 18. For the final 6 weeks of the intervention participants progressed to perform each exercise to set end point of MF followed by a drop set whereby the load was reduced by ~5 kg and an additional set continued to the point of MF was performed immediately upon completion of the first. Load was progressed for each group by 2–10% in the next session if participants could achieve greater than 12 repetitions before reaching the defined set end point for their current period of training (in the case of the final 6 weeks this applied to the first set performed to momentary failure). After the 25-week intervention participants wishing to continue performing resistance training unsupervised were given access to the training facility and allowed to train without direct supervision.

### 2.6. Data Analysis

Independent variables for analyses in the present study were time (before intervention, after intervention, and at follow-up) and also whether participants did, or did not, continue with unsupervised resistance training during the follow-up period. Dependent variables were strength, body composition, physical function, and perceived wellbeing and function. Assumptions of normality were examined using a Shapiro-Wilk test and assumptions of equality of variance examined using Mauchly's test for sphericity. Strength and body composition data met assumptions of normality of distribution and equality of variance so repeated measures analysis of variance (ANOVA) was used to examine effects by time (before intervention, after intervention, and at follow-up). Post hoc pairwise comparisons using a Bonferroni adjustment were conducted comparing preintervention to postintervention period (encompassing the intervention period), postintervention to follow-up period (encompassing the follow-up period), and preintervention to follow-up period (encompassing the whole study). Physical function data met assumptions of normality of distribution and so paired samples* t*-tests were used to examine effects by time (before to after intervention). Questionnaire data did not meet assumptions of normality of distribution and so a nonparametric Friedman test was used to examine effects by time (before intervention, after intervention, and at follow-up). Post hoc pairwise comparisons using Wilcoxon signed ranks tests were conducted comparing pre- to postintervention period (encompassing the intervention period), postintervention to follow-up period (encompassing the follow-up period), and preintervention to follow-up period (encompassing the whole study). Between groups comparisons were made for absolute changes in strength, body composition, and questionnaire data using independent samples* t*-tests and Mann–Whitney* U* tests (follow-up minus postintervention period) for the follow-up period comparing those who either did or did not continue with unsupervised RT. Further, 95% confidence intervals (CI) and within-participant ESs (*d* = *μ*_change_/*σ*_change_; small = 0.20–0.49, moderate = 0.50–0.79, and large = ≥0.80) were calculated for changes in strength, body composition, and physical function across the intervention (post- minus preintervention period) period. Statistical analysis was performed using SPSS statistics computer package (versus .23; IBM, Portsmouth, UK) and *p* ≤ 0.05 set as the limit for statistical significance.

## 3. Results

### 3.1. Strength

**Figure 2:**
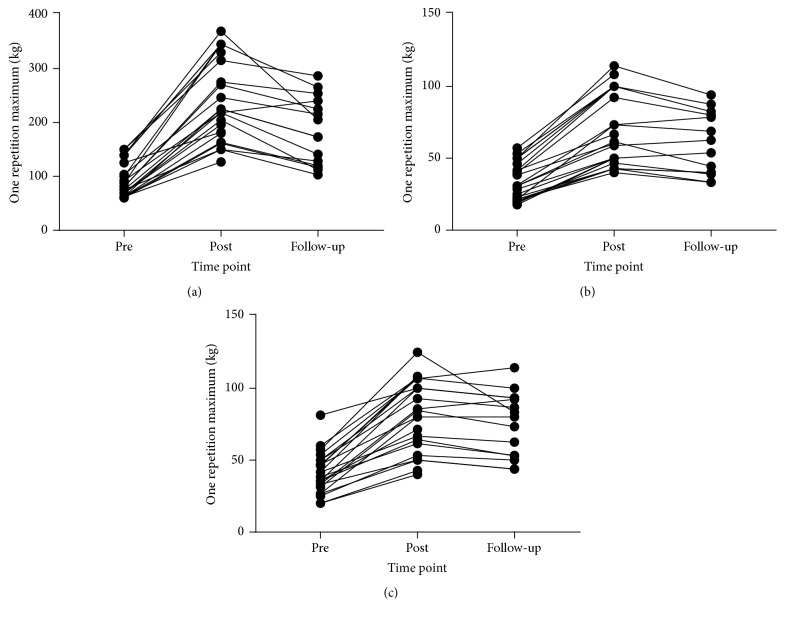
One repetition maximum at preintervention, postintervention, and follow-up period: (a) leg press, (b) chest press, and (c) row.

Descriptive data for all strength outcomes and all time points and 95% CIs and ESs for changes are reported in Table 1.  Figure 2 also shows individual responses at each time point for strength measures. Repeated measures ANOVA revealed significant effects by time for leg press 1RM (*F*_(2,26)_ = 88.876,* p* < 0.001), chest press 1RM (*F*_(2,26)_ = 63.577,* p* < 0.001), and seated row 1RM (*F*_(2,26)_ = 48.750,* p* < 0.001). Post hoc pairwise comparisons revealed significant differences between pre- and both postintervention and follow-up time points for leg press 1RM (*p* < 0.001), chest press 1RM (*p* < 0.001), and seated row 1RM (*p* < 0.001). Significant differences between postintervention and follow-up time points were also found for leg press 1RM (*p* < 0.001) and chest press 1RM (*p* = 0.017).

Independent samples* t*-tests for changes over the follow-up period revealed no significant differences between those who chose to continue with unsupervised training compared with those who did not for change in leg press 1RM (*t*_(21)_ = −1.150,* p* = 0.263; −98.38 kg versus −154.74 kg, resp.), change in chest press 1RM (*t*_(21)_ = −0.522,* p* = 0.607; 30.48 kg versus −38.99 kg, resp.), or change in seated row 1RM (*t*_(21)_ = −1.412,* p* = 0.173; −22.81 kg versus −46.64 kg, resp.).

### 3.2. Body Composition

**Figure 3:**
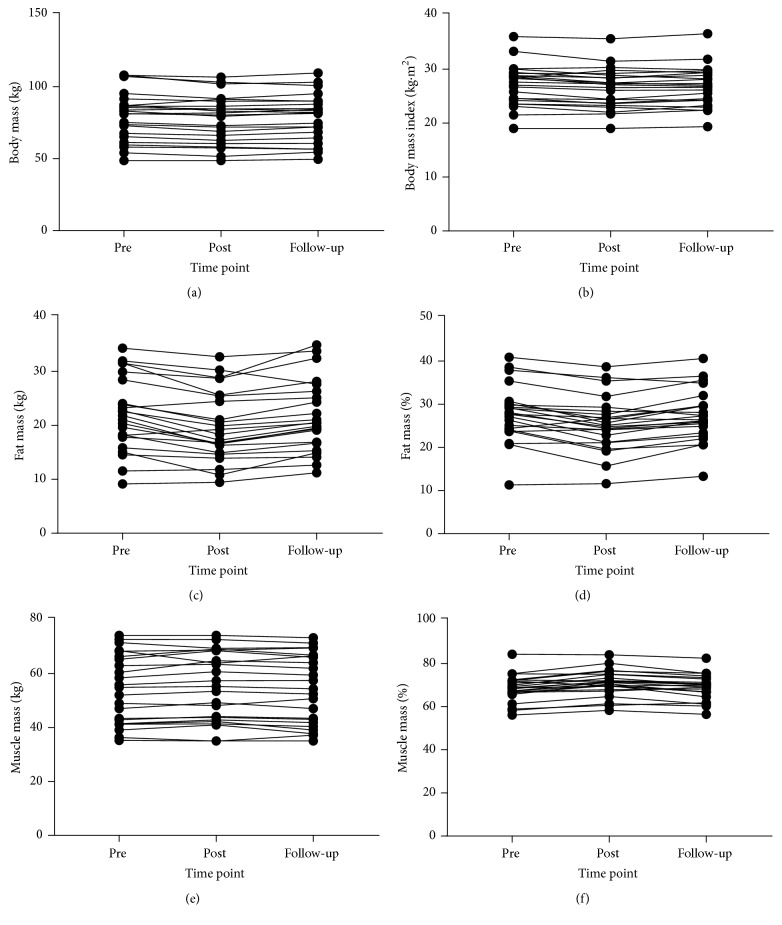
Body composition at preintervention, postintervention, and follow-up: (a) body mass, (b) body mass index, (c) fat mass, (d) fat percentage, (e) muscle mass, and (f) muscle percentage.

Descriptive data for all body composition outcomes and all time points and 95% CIs and ESs for changes are reported in Table 1.  Figure 3 shows individual responses at each time point for body composition measures. Repeated measures ANOVA revealed significant effects by time for BMI (*F*_(2,26)_ = 3.654,* p* = 0.040), fat mass (*F*_(2,26)_ = 9.752,* p* = 0.001), fat percentage (*F*_(2,26)_ = 10.120,* p* = 0.001), and muscle percentage (*F*_(2,26)_ = 10.543,* p* < 0.001), but not body mass (*F*_(2,26)_ = 3.175,* p* = 0.058), or muscle mass (*F*_(2,26)_ = 3.940,* p* = 0.086). Post hoc pairwise comparisons revealed significant differences between pre- and postintervention for fat mass (*p* = 0.003), fat percentage (*p* = 0.004), and muscle percentage (*p* = 0.007). Significant differences between postintervention and follow-up time points were also found for fat mass (*p* = 0.005), fat percentage (*p* = 0.006), and muscle percentage (*p* = 0.004).

Independent samples* t*-tests for changes over the follow-up period revealed no significant differences between those who chose to continue with unsupervised training compared with those who did not for change in body mass (*t*_(21)_ = 0.791,* p* = 0.438; 0.65 kg versus 1.22 kg resp.), change in BMI (*t*_(21)_ = 1.458,* p* = 0.160; 0.09 kg·m^2^ versus 0.45 kg·m^2^, resp.), change in fat mass (*t*_(21)_ = −0.931,* p* = 0.363; 2.13 kg versus 1.47 kg, resp.), change in fat percentage (*t*_(21)_ = −0.671,* p* = 0.510; 2.04% versus 1.54%, resp.), change in muscle mass (*t*_(21)_ = 1.161,* p* = 0.259; −1.02 kg versus −0.24 kg, resp.), and change in muscle percentage (*t*_(21)_ = 0.786,* p* = 0.440; −2.03% versus −1.45%, resp.).

### 3.3. Function

**Figure 4:**
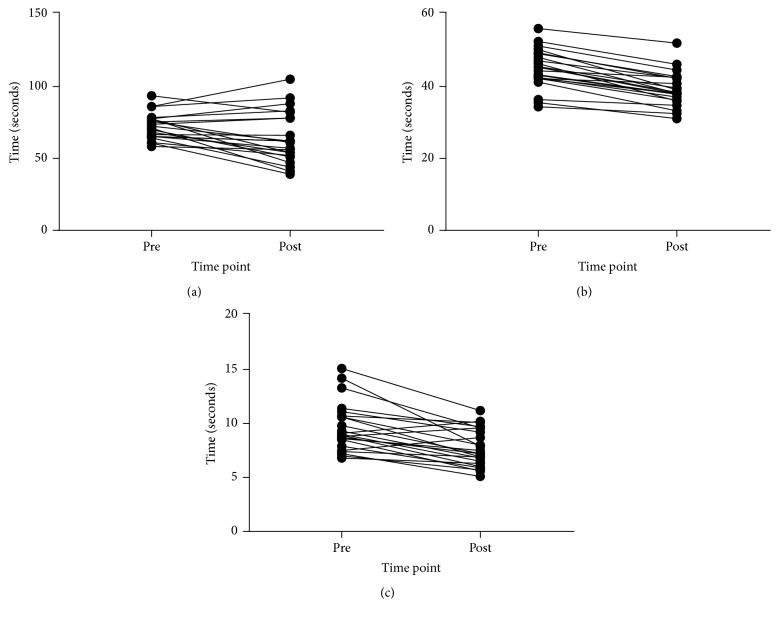
Functional test times at preintervention and postintervention period: (a) stair climb time, (b) parkour time, and (c) chair rise time.

**Figure 5:**
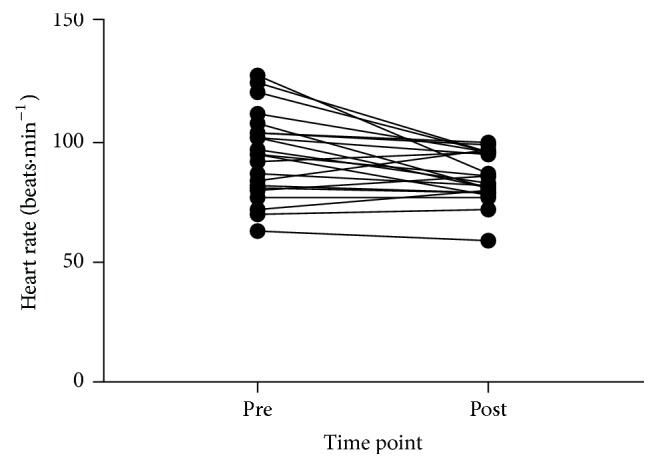
Resting heart rate at preintervention and postintervention period.

**Figure 6:**
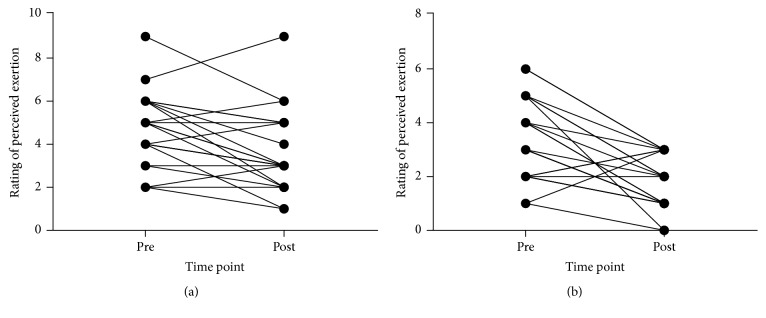
Rating of perceived exertion during functional tests at preintervention and postintervention period: (a) stair climb, and (b) carrying task.

**Figure 7:**
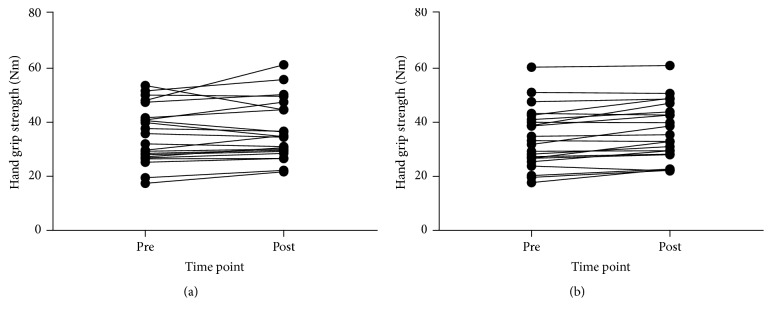
Hand grip strength at preintervention and postintervention period: (a) right and (b) left.

Descriptive data for all function outcomes and all time points and 95% CIs and ESs for changes are reported in Table 2. Figures 4, 5, 6, and 7 show individual responses at each time point for function measures. Paired samples* t*-tests revealed significant differences from pre- to postintervention period for resting heart rate (*t*_(21)_ = 3.096,* p* = 0.005), stair climb time (*t*_(21)_ = 2.858,* p* = 0.009), stair climb RPE (*t*_(21)_ = 3.257,* p* = 0.004), carrying task time (*t*_(22)_ = 10.350,* p* < 0.001), carrying task RPE (*t*_(22)_ = 4.858,* p* < 0.001), chair rise time (*t*_(22)_ = 5.249,* p* < 0.001), and left hand grip strength (*t*_(21)_ = −4.006,* p* = 0.001) but not right hand grip strength (*t*_(21)_ = −1.431,* p* = 0.167).

### 3.4. Questionnaires

Descriptive data for all questionnaire outcomes and all time points and Friedman and Wilcoxon test* p* values are reported in Table 3. Friedman tests revealed significant effects by time for state of health, comparison with other seniors, WHO-1, WHO-2, WHO-3, WHO-4, WHO-5, and perceived ability to accomplish household chores, stair climbing, shopping, gardening, and transport, but not present sporting condition. Post hoc Wilcoxon signed ranks tests revealed significant differences between pre- and both postintervention and follow-up time points for state of health, comparison with other seniors, WHO-1, WHO-2, WHO-3, WHO-4, WHO-5, and perceived ability to accomplish household chores, stair climbing, shopping, gardening, and transport and only pre- and postintervention periods for perceived ability to accomplish sports. Significant differences between postintervention and follow-up time points were also found for WHO-1, WHO-2, WHO-3, WHO-4, and perceived ability to accomplish sports, gardening, and transport.

Mann–Whitney* U* test revealed no significant differences between those who chose to continue with unsupervised training compared with those who did not for change in perceived state of health (*U* = 53.000,* p* = 0.435; 0.00 ± 1.50 pts versus 0.00 ± 1.25 pts, resp.), comparison with other seniors (*U* = 57.000,* p* = 0.648; 0.00 ± 0.50 pts versus 0.00 ± 1.50 pts, resp.), WHO-1 (*U* = 63.500,* p* = 0.927; 0.00 ± 1.00 pt versus 0.00 ± 1.25 pts, resp.), WHO-2 (*U* = 61.000,* p* = 0.832; 0.00 ± 1.00 pt versus −0.50 ± 1.25 pts, resp.), WHO-3 (*U* = 60.500,* p* = 0.784; −1.00 ± 2.00 pts versus −0.50 ± 2.00 pts, resp.), WHO-4 (*U* = 63.000,* p* = 0.927; 0.00 ± 1.00 pt versus −0.50 ± 1.25 pts, resp.), WHO-5 (*U* = 50.000,* p* = 0.376; −0.00 ± 1.50 pts versus −1.00 ± 1.00 pt, resp.), and perceived ability to accomplish household chores (*U* = 57.000,* p* = 0.648; 0.00 ± 1.00 pt versus −0.50 ± 1.00 pt, resp.), stair climbing (*U* = 63.000,* p* 0.927; 0.00 ± 1.50 pts versus 0.50 ± 0.75 pts, resp.), shopping (*U* = 61.000,* p* = 0.832; 0.00 ± 1.00 pt versus 0.00 ± 1.25 pts, resp.), gardening (*U* = 49.000,* p* = 0.343), transport (*U* = 49.000,* p* 0.343; 0.00 ± 1.00 pt versus −0.50 ± 1.50 pts, resp.), and sports (*U* = 61.000,* p* = 0.832; −1.00 ± 1.50 pts versus –1.00 ± 3.25 pts, resp.).

## 4. Discussion

The present study examined the implementation of a 6-month supervised RT intervention introducing increased effort through progressive application of defined set end points. Over the course of the intervention there were significant changes in strength, body composition, function, and wellbeing outcomes. Participants were also followed up for 6 months after intervention. Over the follow-up period body composition changes reverted to baseline values, strength gains were significantly decreased compared to postintervention period but remained significantly higher than baseline, and questionnaire outcomes were mostly maintained. During the follow-up period participants self-selected whether they wanted to continue participating in RT unsupervised (57% of participants continued). When comparing between the group that self-selected continuing with RT and those who did not, there appeared to be no significant differences for changes in any outcome measures over the follow-up period. Despite the initial effectiveness of the RT intervention, these data suggest that continuation of RT unsupervised offered no additional benefit to maintaining intervention induced changes compared with cessation of training.

Improvements in strength across the intervention period were significant and large when considering the ESs. Further, individual responses (Figure 2) revealed that all participants increased in strength though the magnitude of change showed considerable interindividual variability. High effort RT has been shown to produce large improvements in strength in older adults even when employed at a low volume and frequency [38]. The results of the present study further support this whilst employing progressive applications of set end points culminating in use of MF and MF + [drop sets]. This is apparently the first study to employ such RT approach in older adults. Fisher et al. [38] reported within-participant ESs for males and females, respectively, of 2.19 and 2.36 for leg press, 1.59 and 1.59 for chest press, and 2.67 and 2.84 for seated row when considering load progression from beginning to end of ~12- or ~19-week RT intervention, respectively, using sdRM as a set end point. The ESs for change in 1RM in the present study were similarly large (2.66, 2.51, and 2.38 for leg press, chest press, and seated row, resp.) though not larger than that reported by Fisher et al. [38] suggesting that employing higher effort set end points such as MF and MF + [drop sets] may not be necessary to maximise strength gains in older adults. However, strength gains are thought to be highly specific to the task being performed during training [55–57]. As such, for comparative purposes the within-participants ESs for increases in training load across the intervention period for the present study were 3.41, 2.84, and 2.67 for leg press, chest press, and seated row, respectively, suggesting benefits for training to MF and MF + [drop sets] compared with just sdRM for leg press and chest press. However, it should be noted that the present study did not directly compare different RT set end points and so further research should examine this in older populations.

Neither body mass, BMI, nor muscle mass changes across the intervention period were significant; however, there were significant decreases in fat mass and fat percentage as well as increased muscle percentage. Studies applying traditional RT approaches of multiple sets performed to an nRM have reported significant changes in body composition in older adults [58]. However, when higher effort set end points have been used (MF) low volume single set RT is similarly effective [59]. Despite the fact that research by Phillips and Ziuraitis [60] has suggested single set approaches require insufficient energy costs to reduce body fat, the set end points applied in their study were unclear (described as volitional fatigue). Prior studies in young adults using low volume and high intensity of effort approaches have shown similar body composition changes to the present study and these have been ascribed to the higher effort set end points used (i.e., MF and MF+) [48, 61]. Indeed, body composition changes appear greater in older individuals with low volume higher effort interventions compared with higher volume lower effort approaches [62, 63]. Though body compositions changes are possible with participation in high effort RT it is difficult to ascribe the changes reported in the present study purely to the effects of the intervention. Recent work has shown that older adults tend to spontaneously make other lifestyle changes such as improvements in diet including energy intake and increases in non-RT physical activity when initiating and maintaining an RT intervention [64]. As such, RT could act as a first step in public health approaches in the elderly.

With the exception of right hand grip strength, all functional outcomes improved significantly over the intervention period. Questionnaire data further corroborate these improvements suggesting that participants perceived their general state of health, comparison to other older adults, wellbeing, and ability to accomplish many functional tasks were significantly improved after the intervention. As noted, functional ability and risk of falling are associated with strength [15, 16] and as such RT is recommended for improving falls risk, gait ability, and balance in physically frail older adults [17]. The improved functional ability in the present study may therefore have been a result of the significant strength gains produced. Indeed, baseline strength levels are correlated with gait speed and improve with RT [18], and a recent study also reported that the improvements in gait speed as a result of RT are significantly related to gains in lower body strength (*r* = 0.45;* p* = 0.04) [19]. As such, out of curiosity post hoc Pearson's correlation was examined between change in stair climb time and change in leg press 1RM over the intervention period for the present study finding a similar relationship (*r* = 0.48;* p* = 0.003). Despite the apparent relationship between strength and functional ability in older adults the role of improved self-efficacy as a result of participation in the intervention might have impacted upon improved function [65]. Indeed, though functional outcomes were not examined at follow-up, the maintenance of most questionnaire outcomes despite the loss of strength suggested that improvements in perceived function may have been maintained.

During the follow-up period ~57% of participants opted to continue with unsupervised RT, considerably more than those reported by Trappe et al. [44]. The greater maintenance of RT behaviours compared with that reported by Trappe et al. [44] in the present study could be for a number of reasons. Further, though there was a significant loss of 1RM strength for leg press and chest press exercise (though not for seated row) over the follow-up period, strength was still significantly higher than baseline at 6-month follow-up. The same was not the case for body composition changes, all of which returned to baseline levels. The loss of strength and body composition improvements across the follow-up period were not significantly different between those who opted to continue with RT and those who did not. However, though not significantly different, as can be seen from the individual response plots there was considerably greater variation in whether strength was maintained or lost compared with the changes across the intervention period and, further, descriptive statistics suggested that loss of strength was less in the group that continued RT (−98.38 kg versus −154.74 kg, −30.48 kg versus −38.99 kg, and −22.81 kg versus −46.64 kg for leg press, chest press, and seated row, resp.). This did not appear to be the case for the body composition results though with no clear trend for more favourable outcomes in those who continued RT. The loss of strength despite continued RT is interesting considering that prior research has shown that strength gains can be maintained even with a very low RT dose. As noted Trappe et al. [44] reported that the training effort of participants appeared to drop considerably as evidenced by the low number of repetitions being performed with training loads far lighter than during the initial 12-week intervention. Numerous studies have shown that strength and body composition changes are reduced when training without supervision versus training with supervision [66, 67]. Indeed they often train with lower loads and efforts [68, 69] and it has been suggested they likely avoid training to MF [70]. In the present study, at follow-up participants were training with loads lower, but not substantially so, having reduced them by ~2.4% to ~5.5%. As such it seems likely that effort was reduced during follow-up in the present study by avoidance of set end points resulting in higher effort (i.e., sdRM, MF, and MF+). Indeed, the majority of participants who continued training reported at follow-up to have switched to performing a multiple set (~3 sets) program performed to nRM.

Though reduced, it may be that strength gains remain elevated above baseline levels despite reduced effort in continued RT. However, whether given sufficient time participants strength would return to baseline values, despite continued RT without application of sufficient effort by application of appropriate set end points, is a question of importance. If this were the case, efforts to implement behaviour change including participation in RT unsupervised in addition to employment of initial supervised RT in older adults might be considered to represent a costly and ineffective public health approach. Previous studies suggested that older adults lose strength and power faster than young adults [71]. Fiatarone et al. [18] reported 32% strength loss after only 4 weeks of detraining. Moreover, Cadore et al. [72] showed that 1/3 of the participants (older adults with dementia and severe functional loss) died in the 6 months following interruption of a RT intervention. Therefore, it seems that unsupervised low volume resistance training might not be sufficient to overcome this tendency. However, there was also no significant differences between either of the groups for changes in wellbeing outcomes from the questionnaire data the improvements of which were maintained at follow-up. This suggests that the initial supervised intervention may have at least had some lasting effects upon perceived wellbeing which were maintained irrespective of whether unsupervised RT was continued, even if objective outcomes were not maintained. As such, longer term trials are needed to examine the long term effects of initial RT and efficacy of continued participation after initial supervised RT.

## 5. Conclusions

The present study shows that a 6-month supervised RT intervention employing progressive application of high effort set end points is well tolerated and is effective in improving strength, body composition, function, and wellbeing in older adults. Compared with prior studies, a considerable proportion of participants (~57%) opted to continue with RT unsupervised after completion of the intervention. Strength and body composition outcomes were generally reduced at 6-month follow-up, though strength remained above baseline, and wellbeing outcomes were maintained. However, there appeared to be no significant effect upon the degree of loss of improvements whether participants continued, or did not, with RT unsupervised at follow-up. This may be due to the likely reduced effort employed during unsupervised RT. As such, future work should examine approaches to ensure maintenance of initial RT outcomes in older adults when transferring to unsupervised continuation of RT.

## Figures and Tables

**Table 1:** Strength and body composition preintervention, postintervention, and follow-up descriptive data (mean ± SD) and intervention and follow-up period change (Δ).

Measure	Preintervention (mean ± SD)	Postintervention (mean ± SD)	Follow-up (mean ± SD)	Intervention period change (Δ)		Follow-up period change (Δ)	
				95% CIs	ES	95% CIs	ES
*Strength *							
Leg press 1RM (kg)	92.03 ± 31.95	242.86 ± 76.60^a^	183.96 ± 61.91^a,b^	126.29 to 175.35	2.66	−173.64 to −72.13	−1.04
Chest press 1RM (kg)	33.85 ± 12.83	70.60 ± 25.23^a^	59.831 ± 21.83^a,b^	30.42 to 43.09	2.51	−50.68 to −17.10	−0.90
Seated row 1RM (kg)	40.76 ± 14.33	81.99 ± 24.74^a^	74.86 ± 22.46^a^	33.75 to 48.72	2.38	−50.91 to −15.43	−0.81
*Body composition *							
Body mass (kg)	79.19 ± 16.84	77.54 ± 16.44	78.43 ± 16.41	−2.59 to −0.71	−0.76	0.16 to 1.63	0.52
BMI (kg·m^2^)	27.04 ± 3.80	26.49 ± 3.72	26.73 ± 3.71	−0.87 to −0.25	−0.79	−0.01 to 0.51	0.42
Fat mass (kg)	22.06 ± 6.80	19.74 ± 6.44^a^	21.58 ± 6.61^b^	−3.16 to −1.47	−1.19	1.11 to 2.57	1.09
Fat percent (%)	27.90 ± 6.43	25.52 ± 6.30^a^	27.34 ± 6.00^b^	−3.30 to −1.47	−1.13	1.07 to 2.58	1.04
Muscle mass (kg)	54.27 ± 12.77	54.94 ± 12.72	54.27 ± 12.77	−0.14 to 1.48	0.36	1.37 to 0.02	−0.42
Muscle percent (%)	68.47 ± 6.13	70.78 ± 6.00^a^	69.02 ± 5.73^b^	1.45 to 3.20	1.15	−2.53 to −1.02	−1.02

Note: a indicates significant difference (*p* ≤ 0.05) with post hoc pairwise comparison of pre- compared with postintervention/follow-up period; b indicates significant difference (*p* ≤ 0.05) with post hoc pairwise comparison of postintervention compared with follow-up period.

**Table 2:** Function pre- and postintervention descriptive data (mean ± SD) and intervention and follow-up period change (Δ).

Measure	Preintervention (mean ± SD)	Postintervention (mean ± SD)	Intervention period change (Δ)	
			95% CIs	ES
*Physical function *				
Resting heart rate (beat·min^−1^)	94.59 ± 17.82	85.68 ± 10.33^a^	−14.95 to −3.05	−0.64
Stair climb time (seconds)	72.25 ± 9.00	64.27 ± 17.74^a^	−13.91 to −2.44	−0.59
Stair climb rating of perceived exertion (0–10)	4.68 ± 1.73	3.59 ± 1.89^a^	−1.70 to −0.30	−0.67
Carrying task time (seconds)	44.96 ± 5.34	39.19 ± 4.71^a^	−6.70 to −4.49	−2.16
Carrying task rating of perceived exertion (0–10)	3.35 ± 1.50	1.74 ± 1.00^a^	−2.35 to −0.92	−1.01
Char rise time (seconds)	9.63 ± 2.25	7.73 ± 1.73^a^	−2.66 to −1.09	−1.09
Right hand grip strength (kg)	35.34 ± 10.52	36.70 ± 10.91	1.17 to 3.63	0.87
Left hand grip strength (kg)	34.01 ± 10.95	36.41 ± 10.81^a^	−0.62 to 3.33	0.31

Note: a indicates significant difference (*p* ≤ 0.05) for paired samples *t*-test of pre- compared with postintervention/follow-up period; b indicates significant difference (*p* ≤ 0.05) for paired samples *t*-test of postintervention compared with follow-up period.

**Table 3:** Questionnaire preintervention, postintervention, and follow-up descriptive data (median ± IQR), and Friedman and Wilcoxon signed ranks test results (*p*).

Measure	Preintervention (median ± IQR)	Postintervention (median ± IQR)	Follow-up (median ± IQR)	Friedman test (*p*)	Wilcoxon signed ranks test (*p*)		
					Preintervention to postintervention comparison	Preintervention to follow-up comparison	Postintervention to follow-up comparison
*Questionnaire data*							
State of heath	3.00 ± 0.00	4.00 ± 2.00^a^	4.00 ± 1.00^a^	<0.001	<0.001	0.001	0.499
Comparison with other older adults	3.00 ± 2.00	4.50 ± 1.00^a^	4.00 ± 1.00^a^	<0.001	<0.001	0.005	0.084
Sporting condition	3.00 ± 2.00	4.00 ± 1.00^a^	3.50 ± 2.00^b^	0.113	0.006	0.744	0.020
WHO-1	3.00 ± 2.00	4.00 ± 1.00^a^	4.00 ± 0.00^a,b^	<0.001	<0.001	0.007	0.010
WHO-2	3.00 ± 1.00	4.00 ± 1.25^a^	4.00 ± 1.00^a,b^	<0.001	<0.001	<0.001	0.019
WHO-3	3.00 ± 1.25	4.00 ± 1.00^a^	4.00 ± 1.00^a,b^	<0.001	<0.001	0.003	0.003
WHO-4	3.00 ± 2.00	5.00 ± 1.25^a^	4.00 ± 2.00^a,b^	<0.001	<0.001	<0.001	0.017
WHO-5	3.00 ± 1.25	4.00 ± 1.00^a^	4.00 ± 0.25^a^	<0.001	<0.001	<0.001	0.090
Perceived ability to accomplish							
Household chores	3.00 ± 1.25	4.00 ± 1.00^a^	4.00 ± 1.00^a^	<0.001	<0.001	0.002	0.071
Stair climbing	3.00 ± 2.00	4.00 ± 1.00^a^	4.00 ± 2.00^a^	<0.001	<0.001	<0.001	0.589
Shopping	3.00 ± 2.00	4.00 ± 1.00^a^	4.50 ± 2.00^a^	<0.001	<0.001	<0.001	0.327
Gardening	3.00 ± 2.00	5.00 ± 1.00^a^	4.00 ± 2.00^a,b^	<0.001	<0.001	0.001	0.021
Transport	3.00 ± 1.25	4.00 ± 1.00^a^	3.50 ± 1.00^a,b^	<0.001	<0.001	0.001	0.003

Note: a indicates significant difference (*p* ≤ 0.05) for Wilcoxon signed ranks test for pre- compared with postintervention/follow-up period; b indicates significant difference (*p* ≤ 0.05) for Wilcoxon signed ranks test for postintervention compared with follow-up period.
